# Metasurface Design for Dual-Mode Sensors Based on Broken Symmetry Structure

**DOI:** 10.3390/nano15090687

**Published:** 2025-04-30

**Authors:** Rundong Yang, Minjing Dai, Yihao Zhao, Xiangfu Wang

**Affiliations:** 1 College of Electronic and Optical Engineering & College of Flexible Electronics (Future Technology), Nanjing University of Posts and Telecommunications, Nanjing 210023, China; 2 Key Laboratory of Radio Frequency and Micro-Nano Electronics of Jiangsu Province, Nanjing 210023, China

**Keywords:** dual mode, broken symmetry, SPG resonant cavity, FEM

## Abstract

Dual-mode sensors are currently facing difficulties in achieving independent sensing of parameters as well as low sensitivity. In this paper, we propose a dual-mode sensor using the finite element method (FEM) based on a coupled silver–PDMS–gold (SPG) cavity. We coupled a square ring resonant cavity with a double-ring resonant cavity structure, thus identifying a unique resonant cavity structure. The square ring resonator is made of silver and a double-ring resonant cavity filled with PDMS. Our proposed SPG cavity can independently achieve temperature and refractive index sensing. The SPG cavity enables us to obtain the highest biosensing sensitivity of about 1030 nm/RIU and the highest temperature sensitivity of about 216 pm/K. In addition, SPG cavities have excellent tolerances for geometric parameters. Our results provide new methodologies for metasurface design for dual-mode sensing.

## 1. Introduction

A metasurface is an artificial structure with customizable electromagnetic properties that are superior to the limits of natural materials. Recent advances in metasurface-based terahertz device engineering have resulted in functional components such as solar absorbers [1,2,3], optical sensors [4,5,6], polarization modulators [7,8,9], and a number of nonlinear optical devices [10,11,12]. Scientists have also proposed thermally induced tunable terahertz metamaterials [13]. Metasurfaces have a wide range of applications in optical sensing, clinical diagnostics, and biomolecular detection [14,15]. However, most metasurface-based sensors operate in a single sensing mode, typically limited to refractive index (RI) detection [16]. This leads to the sensors having weak anti-interference capabilities, which limits their flexibility and adaptability in practical applications and makes it difficult to meet the specified requirements under various environmental conditions.

In recent years, various design ideas for dual-mode sensors have been proposed to address the above problems. In 2020, Mehdi Aslinezhad proposed a high-sensitivity, high-resolution RI and temperature sensor based on a square ring structure [17], but it has a single absorption peak, which is strongly affected by environmental disturbances, and it has difficulty in achieving accurate sensing. Later, Chao et al. designed a silver-shelled square prism periodic array to enhance uniform excitation distribution in the near-infrared region and further optimize the RI and temperature sensing performance, but the precision optical prisms are costly to process and require specific angle and surface roughness control [18]. Similarly, there is also a dual-mode sensor device with significantly increased RI and temperature, which was designed using materials such as InSb [19,20] and graphene [21]. Despite advances in recent studies, the reported sensitivities still leave room for improvement, and there are even fewer devices that can realize the independent sensing functions of temperature and RI. If independent sensing of RI and temperature is not possible, the cross-sensitivity of the two variations results in false signals that will misinterpret environmental disturbances. Therefore, the development of new optical sensors has become a hot topic of discussion among scientists.

In the study reported in this paper, a metasurface sensor supporting independent sensing of temperature and RI was designed. By coupling a square ring resonant cavity with broken symmetry and a double-ring resonant cavity structure, the design identifies a unique silver–polydimethylsiloxane–gold (SPG) resonant cavity. The double-ring resonant cavity filled with polydimethylsiloxane (PDMS) supports temperature sensing. By breaking the symmetry of the silver square ring structure, the single mode splits into separate modes. This leads to higher-order resonances being excited and enhanced mode coupling. This allows for the introduction of new absorption peaks in the spectrum to support the resonance peaks used for RI sensing. The molecular concentration can be quantitatively analyzed by measuring the RI change, and the independent sensing of temperature and RI allows for better immunity to interference. Numerical simulations show that the sensitivities of RI sensing and temperature sensing are about 1030 nm/RIU and about 216 pm/K, respectively. 

## 2. Materials and Methods

To achieve independent sensing of RI and temperature, we tried to find structures where the resonance peak positions are separated from each other, thus avoiding spectral overlap. The symmetry-breaking square ring resonator and the double-ring resonator can be effectively satisfied. In addition, they have less influence on each other, so the purpose of independent sensing can be effectively achieved. 

**Figure 1:**
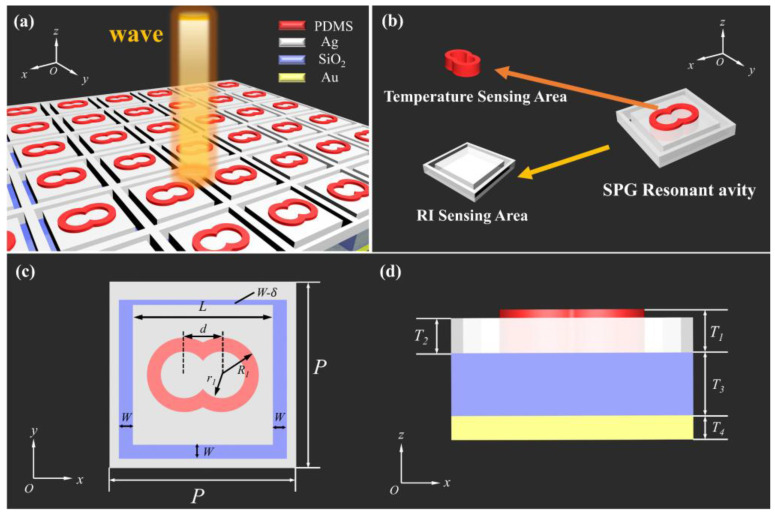
(**a**) Array model of our proposed SPG resonant cavity. (**b**) Unit structure diagram of a single SPG resonant cavity and its corresponding sensing module. (**c**,**d**) Unit plan structure diagram of an SPG resonant cavity.

Figure 1a reveals the structure of the proposed SPG resonant cavities, which consist of periodic double-ring resonators at the top and square ring resonators. The double-ring resonator is filled with PDMS. The function of the PDMS’s RI *n_P_* and the temperature *T* of the PDMS can be expressed as follows [22]:

(1) $$n_{P}=-4.5\times 10^{-4}\cdot T+1.4176$$

The square ring resonator consists of Ag. The symmetry of the SPG can be broken by decreasing the width *W* of the square ring in the square ring resonator by the *δ* length to obtain the resonance peak. The SPG resonator cavity has a SiO_2_ layer in the middle and a gold substrate layer.

The SPG resonant cavity structure has a period of *P* = 800 nm in both the *x* and *y* directions. Figure 1c,d illustrate the structural diagram of the SPG resonant cavity unit and its corresponding variables. Considering the coupling between the parts of the resonator, we set the outer ring radius and inner ring radius of the double-ring resonator to *R*_1_ = 120 nm and *r*_1_ = 80 nm, respectively. The spacing between the double rings is *d* = 140 nm, and the length of the inner square side of the square ring resonator is *L* = 550 nm. The width of the square ring is set as *W* = 50 nm. The height of the double ring resonator we designed is *T*_1_ = 150 nm. It is embedded in the square ring resonator with a height of *T*_2_ = 80 nm. The thicknesses of the SiO_2_ dielectric layer and the Au substrate are *T*_3_ = 500 nm and *T*_4_ = 50 nm, respectively.

In this work, we utilize the finite element method (FEM) for our calculations. The RI of SiO_2_ was set to 1.45 [23]. The thermo-optic coefficient (*TOC*) value can be used as an important indicator of the properties of temperature sensing materials, which is defined as *dn*/*dT* [24]. To select a suitable temperature sensing material, we compared the *TOC* values of ethanol (C_2_H_5_OH), SiO_2_, and silicon (Si). The *TOC* value of C_2_H_5_OH is about −3.94 × 10^−4^ [25], the *TOC* value of Si is about 1.84 × 10^−4^, and the *TOC* value of SiO_2_ is about 1 × 10^−5^ [26]. Among these sensing materials, the sensing sensitivity of PDMS is much higher than the other three materials. Therefore, it can obtain a sensitivity that far exceeds that of the other sensing media. We chose PDMS as the temperature-sensing material for the double-ring resonator. The material properties of gold and Ag were obtained from data in the experimental tables of Werner et al. and calculated data tables of Yang et al., respectively [27,28]. The absorption on the SPG cavity can be expressed as *A* = 1 – *T* − *R*. Since the *T* of the proposed SPG is almost zero, which gives an absorption rate *A* = 1 – *R*. 

To evaluate the sensing performance of the sensor, some performance metrics are used: sensitivity (*S*), figure of merit (*FOM*), full width at half maximum (*FWHM*), and quality factor (*Q*). *S* is mathematically expressed as follows:

(2) $$S_{n}=\frac{∆\lambda }{∆n},\;S_{T}=\frac{∆\lambda }{∆t}$$

where ∆*λ*, ∆*n*, and ∆*T* are the changes in wavelength, RI, and temperature, respectively. *FOM* can be calculated from the following equation:

(3) $$FOM=\frac{S}{FWHM}$$

The *Q* factor can be expressed as follows:

(4) $$Q=\frac{\lambda_{e}}{FWHM}$$

where *λ_e_* is the resonant wavelength.

## 3. Results and Discussions

**Figure 2:**
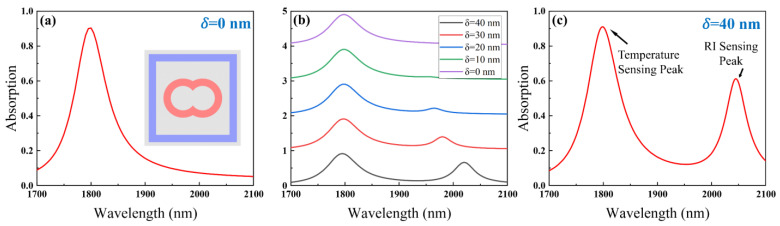
(**a**) Absorption spectra of the SPG resonator cavity with unbroken symmetry (*δ* = 0 nm). (**b**) Absorption spectra of the SPG resonator with different *δ*. (**c**) Absorption spectra of the SPG resonator cavity with broken symmetry (*δ* = 40 nm).

We couple a double-loop structure with a square-loop structure with broken symmetry. Compared to the symmetric structure, the resonant wavelength is more sensitive to the variation of the surrounding RI (Δ*n*) after the symmetry breaking. By combining the advantages of these two structures, we can obtain a dual-mode sensor with better performance. Double-ring resonator and symmetry-broken square-ring resonator excitation of absorption peaks at different wavelengths. Therefore, we coupled these two structures, and independent sensing of temperature and RI can be realized by monitoring the offset of each resonance peak. Figure 2a illustrates the absorption spectrum of the unbroken–symmetric SPG resonator at 1700–2100 nm plane wave incidence. In this case, only one absorption peak can be excited. Interestingly, by breaking the symmetry of the square ring structure, we can obtain another sharp absorption peak. We describe the absorption spectra of the SPG resonant cavity at different *δ* as shown in Figure 2b. As the broken symmetry strengthens, the absorption peaks increase and the absorption peaks become sharper. By breaking the symmetry of the square ring structure, we can introduce new absorption peaks in the absorption spectrum, as shown in Figure 2c. 

**Figure 3:**
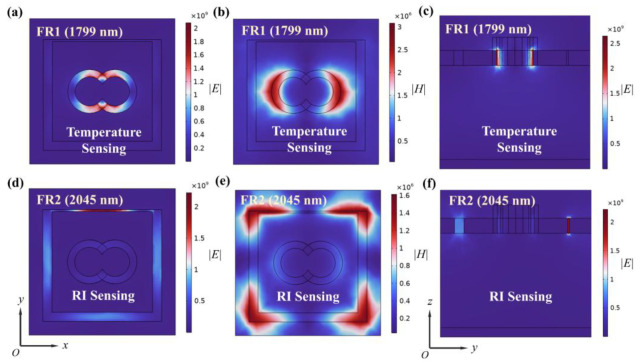
The (**a**) electric field *|E|* distribution in the xoy plane, (**b**) magnetic field *|H|* distribution in the xoy plane, and (**c**) magnetic field *|E|* distribution in the yoz plane of the SPG resonant cavity at the FR1 wavelength. The (**d**) electric field *|E|* distribution in the xoy plane, (**e**) magnetic field *|H|* distribution in the xoy plane, and (**f**) magnetic field *|E|* distribution in the yoz plane of the SPG resonant cavity at the FR2 wavelength.

Additionally, we calculated the distributions of the electric field *|E|* and magnetic field *|H|* of the SPG at the resonant wavelength. The electrons undergo collective oscillations driven by the incident light to form surface-isolated excitations, polarized excitations. Figure 3a,b shows the electric field and magnetic field distributions of the SPG resonator at the FR1 resonant wavelength, respectively. At the FR1 resonant wavelength, the electric field is mainly concentrated in the double-ring resonator, while the magnetic field is distributed between the double-ring resonator and the square ring resonator. The electric field distribution in the yoz plane in Figure 3c also indicates that the double-ring resonator plays a major role when applied to the absorption peak for temperature sensing. At the FR2 resonant wavelength, the electric field is concentrated in the square ring resonator, and the strength of the electric field is greater where the symmetry is broken, as shown in Figure 3d. The magnetic field is distributed in the corners of the square ring resonator, as shown in Figure 3e. Applied to the absorption peaks of RI sensing, the strong electric field in the damaged square ring resonator is gathered as shown in Figure 3f, and it is easy to see that the square ring resonator contributes a lot to the formation of the absorption peaks at this time. These phenomena demonstrate the existence of both electric and magnetic dipole resonances at both FR1 and FR2 wavelengths. Thus, the interaction between electrical and magnetic resonance leads to the formation of absorption peaks [29].

Next, we investigated the RI sensing performance as well as the temperature sensing performance of the SPG resonant cavity, respectively.

**Figure 4:**
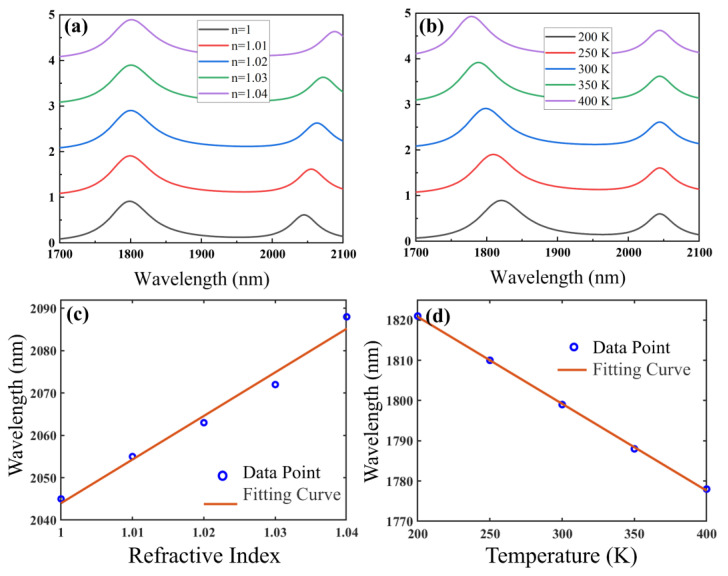
The SPG resonant cavity’s (**a**) absorption spectrum as a function of RI and (**b**) absorption spectrum as a function of temperature, (**c**) FR2 peak displacement as a function of RI and its linear fit. (**d**) FR1 peak displacement as a function of temperature and its linear fit.

The spectral redshift of FR2 in the RI range of 1.00–1.04 is about 43 nm, as shown in Figure 4a, and we then linearly fit a plot of FR2 peak migration versus RI, as shown in Figure 4c. Through linear fitting, we can conclude that the *S_n_* of FR2 is about 1030 nm/RIU, which is better than that of some recent single-mode RI sensors, including the 765.4 nm/RIU achieved by Reiter et al. [30], the 556.9 nm/RIU achieved by Zhu et al. [31], and the 578.3 nm/RIU achieved by Zhao et al. [32]. Additionally, since the *FWHM* of FR2 is about 41.9 nm, we also calculated that the *FOM* and *Q* of FR2 for sensing are about 24.6 RIU^−1^ and 48.8, respectively. 

The 43 nm of FR1 is blue-shifted when the temperature is increased from 200 K to 400 K, as shown in Figure 4b. In Figure 4d, we linearly fit the resonance wavelength of the resonant FR1 to the resonance peaks at different temperatures to determine the temperature dependence of the resonance peaks. The *S_T_* of FR1 was determined to be about 216 pm/K. This result is better than the 59.5 pm/K measured by Zhao et al. [33] and 34 pm/K measured by Wang et al. [34]. Additionally, since the *FWHM* width of FR1 is about 65.5 nm, the *FOM* and *Q* factors used for temperature sensing are about 0.0033 K^−1^ and 27.5, respectively. Therefore, we achieved the temperature sensing performance of our proposed SPG resonance cavity.

**Figure 5:**
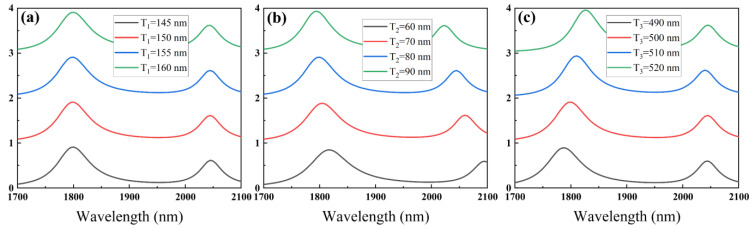
Absorption spectra of the proposed cavity as a function of (**a**) *T*_1_, (**b**) *T*_2_, and (**c**) *T*_3_, respectively.

Finally, we discuss the effect of the geometric parameters of the SPG resonant cavity on the sensing characteristics. Figure 5 gives the absorption spectra of the SPG resonant cavity for three parameters, *T*_1_, *T*_2_, and *T*_3_, respectively, varied within the design values. In Figure 5b, the increase in the thickness of the square ring resonator *T*_2_ leads to a blue shift of the resonant wavelengths FR1 and FR2. As shown in Figure 5c, the FR1 has a significantredshift with the increasing thickness of the SiO_2_ layer. Unlike the two parameters *T*_2_, and *T*_3_, the increase in the thickness of the double-ring resonator *T*_1_ has little effect on the absorption spectra, as shown in Figure 5a. These results show that small changes in geometric parameters do not lead to significant changes in sensing characteristics, which helps to overcome the effects of practical tolerances.

To highlight the outstanding performance of our proposed SPG resonator among the equipartitioned excitation multimode sensors, we compared it with our previous work, as shown in Table 1.

From Table 1, we can see that our proposed SPG resonant cavity has excellent sensitivity performance. Its ability to support independent sensing of temperature and RI has further expanded its practical applications. The sensors we have proposed so far, although realizing independent sensing and greatly improved sensitivity, are still deficient in terms of *FOM* and *Q* values. 

## 4. Conclusions

In this paper, we propose a dual-mode sensor driven by a symmetry-broken structure based on Ag-PDMS-Gold. We couple a square ring resonant cavity with a double-ring resonant cavity structure to identify a unique SPG resonant cavity structure, which enables independent sensing of temperature and RI parameters. The SPG resonant cavity structure allows us to obtain the highest biosensing sensitivity of about 1030 nm/RIU and the highest temperature sensitivity of about 216 pm/K. The SPG resonant cavity structure also ensures high excellent geometric parameter tolerances. The RI is sensed with an *FOM* of about 24.6 RIU^−1^ and a *Q* of about 48.8, while the temperature is sensed with an *FOM* of about 0.0033 K^−1^ and a *Q* of about 27.5. Our results provide new methodologies for metasurface design for dual-mode sensing.

## Figures and Tables

**Table 1:** Comparison with previous work.

Ref.	*S_T_*	*S_n_*	Sensing Mode	*FOM*	Max *Q*
[35]	/	1435.71 nm/RIU	RI	80 RIU^−1^	/
[36]	/	1100 GHz/RIU	RI	3.832 RIU^−1^	/
[37]	78.7 pm/°C	286.82 nm/RIU	RI and Temperature	/	/
[38]	180 pm/°C	355 nm/RIU	RI and Temperature	/	/
[39]	336 pm/°C	737.71 nm/RIU	RI and Temperature	/	20.5
Our work	216 pm/K	1030 nm/RIU	RI and Temperature	0.0033 K^−1^, 24.6 RIU^−1^	48.8

## Data Availability

Data are contained within the article.
